# Unpacking the ‘Business Model’ for Fortification Initiatives in Low- and Middle-Income Countries: Stakeholder Identified Drivers of Success and Constraints to Progress

**DOI:** 10.3390/ijerph17238862

**Published:** 2020-11-28

**Authors:** Baqir Lalani, Michael Ndegwa, Ben Bennett

**Affiliations:** Natural Resources Institute, Medway Campus, University of Greenwich, Central Avenue, Chatham Maritime, Kent ME4 4TB, UK; M.K.Ndegwa@greenwich.ac.uk (M.N.); Ben.Bennett@greenwich.ac.uk (B.B.)

**Keywords:** industrial fortification, business models, scale, standards, testing

## Abstract

*Background:* Initiatives to tackle micronutrient deficiencies (MNDs) in low-and middle-income countries (LMICs) have increased steadily in recent years. Commodities such as staple foods (e.g., cereals) and condiments (e.g., salt) have been targeted as ‘vehicles’ for industrial fortification through numerous projects and initiatives. However, mixed experiences with delivery, coverage and sustainability have been found. *Methods:* Using an online survey of 71 key stakeholders (from 35 countries) consisting of the public/private sector, academia and civil society, this study sought to unpack the ‘business model’ for fortification initiatives to identify the key drivers of success and constraints faced by stakeholders in LMICs. Bivariate analysis was conducted to identify factors associated with the coverage of the target market and the perceived success and sustainability of fortification initiatives. *Results:* We identified four key factors contributing to the success of fortification initiatives. The first involves the size of the firm. Large firms had a significantly higher (*p* 0.05) self-sustaining index (perceived level of sustainability of the fortification initiative) than smaller sized firms. In addition, a higher perceived success score (*p* 0.05) was associated with non-targeted initiatives compared to those specifically targeted at a certain cohort of the population, further illustrating the benefits of producing at scale. Secondly, a significant relationship was found between whether standards were enacted and the coverage of the target market by the project/firm (*p* 0.05).). The third key factor relates to the ability to source adequate testing for the fortified produce in-house. A positive correlation was found for post-mix in-factory testing and the self-sustaining index (*p* 0.05). Finally, delays to importation and high charges were cited as key constraints to the use of premix. *Conclusions:* We argue therefore that a successful ‘business model’ for industrial fortification initiatives invariably consist of: (i) the involvement of larger sized firms that have the advantage of benefiting from economies of scale; (ii) the availability and application of agreed standards by the producer; (iii) high quality assurance/compliance monitoring (including post-mix testing where relevant), and; (iv) the ability to procure premix in a timely/cost-effective manner. These criteria are likely to be important factors that contribute to the success of fortification initiatives in LMICs.

## 1. Introduction

Food systems in many low and middle income countries (LMICs) are faced with increasing challenges to deliver nutritionally adequate diets including population growth, urbanisation, and climate change [1,2]. Coupled with this, approximately 3 million child deaths per year relate to deficiencies of essential vitamins and minerals such as Vitamin A, D, iron and zinc [1]. Initiatives to tackle micronutrient deficiencies (MNDs) in LMICs have increased steadily in recent years [3]. Commodities such as staple foods (e.g., cereals) and condiments (e.g., salt) have been targeted as ‘vehicles’for industrial fortification (fortification requires suitable food vehicles, i.e., those foods that are widely consumed and therefore have the ability to be reached/accessed by the most amount of people—though does not aim to increase consumption of the product) whereby one or more minerals or vitamins are added to the commonly consumed foods [4]. Benefits from industrial fortification initiatives have been found in LMICs such as improvements to food safety [3] and as with the case of wheat flour in Morocco, where there was a significant reduction in the prevalence of anaemia in children aged 3 to 5 years [5]. Furthermore, a recent systematic review of 80 countries found that flour fortification has had a significant impact on reducing the prevalence of low ferritin among women [6]. In contrast, a number of national fortification initiatives have had limited impact and failed to reach very remote communities or scale due to a myriad of issues including inadequate funding, project design, weak governance and compliance issues [5,7,8]. A lack of research surrounding the drivers of the success and failure of food fortification initiatives includes limited research on the range and effectiveness of the business models (the terms business models and delivery models are used interchangeably throughout the manuscript—we define business model as “A specific combination of resources which through transactions generate value for customers and the organization” [9].) used to deliver the expected fortification results. In recent years, extensive reviews of fortification programmes in LMICs and a global mapping exercise have been conducted [3]. though little has been documented surrounding the views of stakeholders involved in these projects. Recent research has also highlighted that many LMICs can benefit substantially from industrial fortification initiatives, yet these are seldom pursued [2]. This study forms part of a larger research project with the overall purpose of the research project to identify the business models that drive the success or failure of food fortification initiatives. This study, however, seeks to unpack the ‘business model’ of fortification initiatives to better identify what works and where bottlenecks remain in order to guide programme implementation and enhance the sustainability of programme interventions.

### 1.1. Background of Research Project

This research responds to a wider global effort to use the power of food fortification to address key aspects of the Sustainable Development Goals [10] and a widely held belief that fortification is more cost-effective than alternative solutions in reducing micronutrient deficiencies (MNDs). There are global efforts to expand investment, scale and impact using these approaches. For example, the Global Alliance for Improved Nutrition (GAIN), the Flour Fortification Initiative and the Nutrient Foundation. There are also numerous national level, donor, NGO and private sector-led efforts in this field [11]. As part of this, the European Commission (EC) has established the Food Fortification Advisory Services (2FAS) to contribute to strengthening food fortification programmes through collecting and disseminating knowledge and promoting action and commitment at the global and national levels. This effort by major supporters of food fortification has led to a desire to better understand the dynamics and drivers of food fortification sustainability through different business models and approaches.

**Figure 1:**
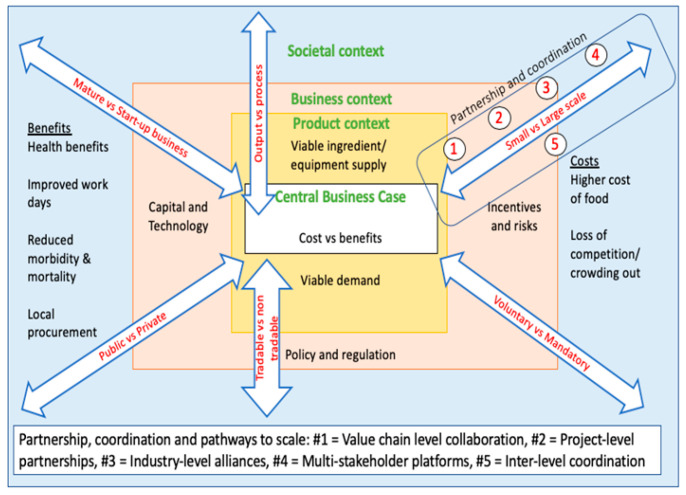
Business viability factors.

The research project was comprised of two phases. These are a review phase and a formative research phase. The initial results of a comprehensive review of literature [12] suggested a framework for the analysis of different business models. This was based on drivers identified by the authors which showed that the scale, technology, incentives, governance, policy and regulation, maturity of business, supply of inputs, demand for finished products and form of intervention (e.g., output vs. process) were all important to success and failure (Figure 1).

### 1.2. Busines Viability Factors Explored

The concept of a central business case is premised on financial viability being at the core of every business model in some form or another. Failure of income to exceed costs results in firm failure. This ‘Central Business Case’ is at the heart of all food fortification efforts. Viability can be assured in many ways ranging from a full subsidy from the state or another actor, to a completely market-based approach with costs being subsumed by non-state actors such as farmers, processors, retailers and/or consumers. The subsidy of food fortification comes in many forms and for many reasons: to promote start-up and adoption; to over-come scaling and investment challenges; to address aspects of market failure and to respond to needs in wider society. States are sometimes ‘fortification takers’ in that they are unable to implement sufficient control over their domestic food policy to manage initiatives and therefore accept policies from private or non-state actors. This is more common in fragile states. Each business format or model is in itself located within a ‘product environment’. For example, fortified cereals compete with substitutes in the same market space, so to compete in this product environment the same business case needs to be made with the viability of supply and demand assured for business success. In this context, managing business risk and competition is important with firms responding to incentives that match these challenges. Fortification programmes are also set in a wider societal context where decisions have to be made between investments that bring about societal benefits, such as improved health, and externalities, including the distorting impacts of subsidies. Added complexity comes from the range of different business starting points: not all fortification initiatives are ‘start-ups’; sectors are governed differently (some highly concentrated and associated, others dispersed and uncoordinated); and approaches to fortification business building vary greatly, ranging from being purely factor driven (e.g., responding to targets) to society driven (e.g., responding to needs, behaviours and trends).

### 1.3. Business Models Defined

Lalani et al., 2019 [12] in a review of the efficacy of the different models used in fortification highlighted four different types: (i) public-led, (ii) private-led, (iii) multi-sector partnerships and (iv) community-led models. Public-sector models are national fortification strategies which typically include mandatory fortification (legislation), enforceable regulation and strong quality assurance and compliance. For example, in Uzbekistan, national fortification programmes have supported millers with social marketing; mandating packaging of flour and subsidised laboratory equipment to improve compliance [5]. Private sector models of fortification follow voluntary fortification and are based on the commercial market development of their products. They may, however, benefit from input from the public sector in the form of incentives (e.g., tax related or equipment/training, etc.). Voluntary fortification has been successfully piloted at a regional scale in India (e.g., vegetable oil) which influenced the government to introduce mandatory legislation [3]. Multi sector partnerships are those which consist of a variety of stakeholders, e.g., public sector bodies, the private sector and civil society organisations. For example, in recent years, national fortification alliances (NFAs) have emerged (e.g., Senegal, Tanzania) [3]. An example of a community model can be found in Senegal where mills were ‘community managed’ businesses with the profits being kept by the operators [13]. As the central business case (Figure 1) shows, depending on the model, ‘food vehicle’, the legislation and country setting can be overlap among various levels of engagement. For example, four levels/pathways have been suggested to scale business engagement in sustainable development [14]. Level 1 refers to cooperation along value chains (with minimum involvement from the private sector towards sustainable development including nutrition). Level 2 relates to project-level partnerships such as linkage with investors, governments and research centres, whilst level 3 is more organised with industry-level alliances and a stronger commitment/organised approach to sustainable development goals). Level 4 consists of multi-stakeholder institution platforms and networks which can be formalised or informal platforms and consist of high commitment to sustainable goals and development. Finally, level 5 consists of coordination between all the different levels (levels are depicted under partnerships and coordination 1–5 in Figure 1) some can be led by business, government or civil society [14].

Building on [12], a conceptual framework in the literature review suggested questions, themes, and areas for further investigation, and these were woven into a question narrative (see Table 1).

Furthermore, this study focusses primarily on the level of regulation, infrastructure and coverage in relation to industrial fortification initiatives [3], has identified ‘key success factors and preconditions’ for large scale fortification, including clear legislation and appropriate standards, as well as the tracking/reporting of the overall coverage of the population using the fortified foods and the need for product quality safety monitoring. Likewise [15], has hypothesised with reference to large scale universal salt iodisation (USI) that four groupings exist: (i) countries with an optimal level of coverage (scaled-up programmes) but where focus on disadvantaged/marginalised groupings needs attention; (ii) countries in scale-up phase, with limited coverage and where the strengthening of value chains will improve the quality/quantity of production (e.g., capacity of producers, quality assurance) and coverage among market segments; (iii) countries where no policies exist and awareness among key stakeholders is paramount (e.g., public, private, civic, academic sectors) and; (iv) fragile states where the enabling environment is not conducive to high coverage rates and exacerbated by governance issues and political/civil strife/natural disasters, etc.

## 2. Methods

Using findings from the desk phase [12] (i.e., literature review), a survey was developed and administered online using SurveyMonkey Inc^®^ (San Mateo, CA, USA). Snowball sampling was used to identify the key stakeholders and those in their own sphere/network from the public/private sectors and civil society across a range of geographies. In addition, existing literature on fortification and biofortification was used to identify those that were involved in the design/implementation of fortification/biofortification projects in academia. In total, 500 stakeholders were contacted with an overall response rate of 21%, i.e., 103 respondents (67 males and 31 females) completed the survey from 39 different countries across sub-Saharan Africa, South Asia and South America). We chose to report on 71 stakeholders (from 35 countries) involved in fortification initiatives for this study. One additional survey response was excluded as it was incomplete. The survey was confidential and respondents were anonymised and only identified by a unique identifier. Ethics approval was granted by the University of Greenwich Ethics Committee. We took a ‘projectised’ approach to analysing the data. This means that it was assumed that many of the individuals had knowledge of specific time-bound projects with defined objectives and results. To encourage greater participation, we asked respondents to focus on only one example where they had the most experience.

The survey drew its themes from the conceptual framework that emerged from the literature review (see Figure 1). Topics including: (i) the types of projects respondents are involved in; (ii) the level of standards/legislation applied; (iii) funding sources of projects, (iv) targeting and (v) premix usage and technology. Additionally, perceptions of, for example, the sustainability, and success of projects, the level of compliance monitoring were also gathered through rating/ranking scales in order to allow for the exploration of bivariate associations and statistical inference. A few open-ended questions were also included to gain stakeholder views on, e.g., reasons for the success of certain projects. Thematic analysis was used to code the data and then search for key themes/sub themes [16].

### 2.1. Data Analysis and Scoring Used

The R software [17] was used to perform non-parametric tests (e.g., Kruskal–Wallis, Wilcoxon, Spearman tests) for inference, given that the data were mainly based on scale/ranking questions [17]. Figures were produced using STATA version 15 (StataCorp., College Station, TX, USA). One-way analysis of variance (ANOVA) was used for the comparison of group means. Several scores/indexes were created to measure the success and sustainability of the fortification initiatives. Likewise, a success index was used to measure the perceived success of the projects stakeholders had been involved in (success index: 1 = failure, 2 = too early to tell 3 = success). Similarly, the sustainability of these projects/business models was also measured on a scale of 1–4 (i.e., 1 refers to a model that is no longer functional or requires 100% subsidy; 2. it requires funds mostly from public subsidy; 3. reliant mostly on the sales of the product; 4. 100% self-sufficient). Finally, the coverage of the target market was also measured (1 = 0%—no production at the end of project; 2 = 0–33% some—limited sustainable production achieved; 3 = 33–66% some—most markets supplied, but not all, 4 = 66–100% most—many markets receiving and using fortified food, but some gaps; 5 = 100%—all domestic food of this type fortified with no subsidy). A full list of the variables used in the analysis is presented in Table 2.

### 2.2. Limitations

This study is not without limitations. Selection bias may be the predominant limitation given the survey design may have only selected those respondents which agreed with fortification and are likely to be more positive than those that are not involved. Potential bias could also relate to the roles stakeholders play in the project, which could have a bearing on their responses/views. The sample size was also small and the universe unknown. Whilst we attempt to explore statistical inference, we do not claim to provide evidence-based solutions. Only a small proportion of the respondents shared their views on the success of fortification initiatives, thus the interpretation of these results (stakeholders’ views section) should be treated with caution.

## 3. Results

The results section is split into three sections. We first explore the description of the sample respondents and funding sources of the respective fortification projects they have been involved with. This considers some of the ‘contextual’ aspects including information about the respondents, their institutional background and the funding sources of the project(s)/businesses that they have been associated with.

In Section 2, we look at different aspects of the chosen business model from the perspective of the drivers identified in Figure 1. These include: firm size, targeting, legislation level; standards and chosen business models, premix and imported elements. The final section investigates the stakeholders’ views on the reasons behind the success of fortification initiatives. A full list of the measures of association/statistical significance between the key characteristics and success measures are found in the Appendix A (See Table A1).

### 3.1. Repondents by Type of Organisation and Main Funding Source (“Context” in Figure 1)

The survey respondents ranged from a variety of backgrounds with a high proportion from international organisations (28%) and public sector bodies (31%). The private sector and academia were also well represented. The majority of respondents were involved in projects relating to fortified cereals and flours (56%) (with a much lower proportion working with projects relating to complementary foods (12%). Of those working in fortified cereals and flours, the majority (over half) were involved in projects relating to the fortification of wheat and/or maize flour. In contrast, for those involved in complementary foods, 52% of respondents were involved in projects that involved mixed porridges and weaning foods (data not shown).

The fortification activities reported by those surveyed were primarily donor-led funded projects (70%) with only a small proportion of those driven by private sector (approximately 20%) or public sector funding in comparison (data not shown). However, public sector funds formed a much higher proportion of the secondary source of funds, which indicates that these projects, on the whole, whilst reliant on donor funding or private sector funding, are likely to be reliant on significant investment from the public sector.

### 3.2. Firm Size, Targeting and Legislation Level (“Scale”, “Mature vs. Start-up” “Voluntary vs. Mandatory” and in Figure 1)

**Figure 2:**
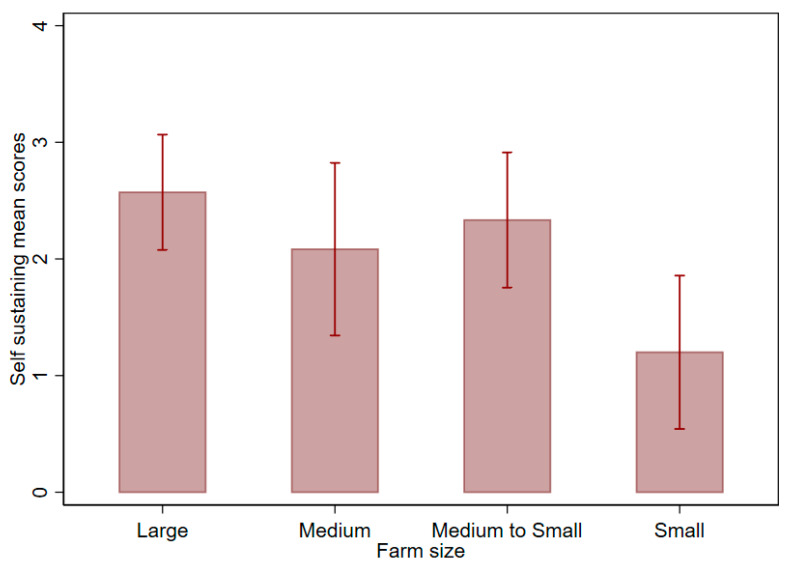
Mean self-sustaining index by firm size (self-sustaining index: 0 = model no longer functional or requires 100% subsidy; 1 = mostly from public subsidy; 2 = mostly from sales; 3 = 100% self-sufficient). Firm size (based on number of employees). Note: error bars = standard errors (for Figure 2, Figure 3 and Figure 4, the truncated axes start at zero for the ease of interpretation).

**Figure 3:**
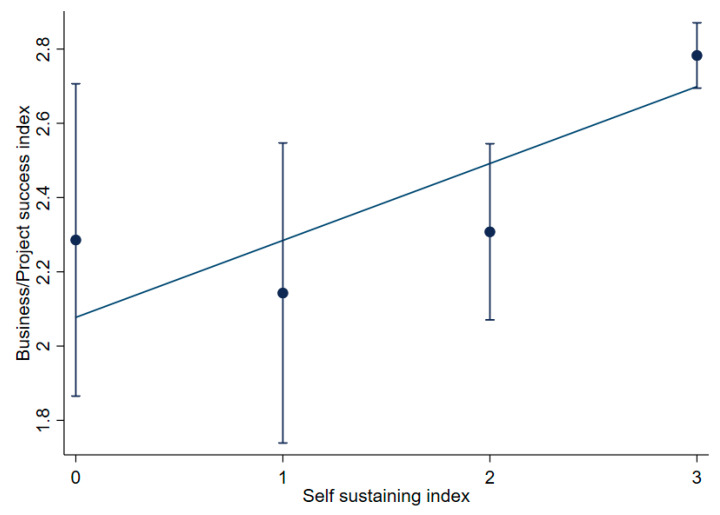
Success index by the self-sustaining index (success index: 1 = failure; 2 = too early to tell; 3 = success—self sustaining index: 0 = model no longer functional or requires 100% subsidy; 1 = mostly from public subsidy; 2 = mostly from sales; 3 = 100% self-sufficient). Note: means = dots; diagonal line = regression line; and error bars = standard errors.

Figure 2 shows the self-sustaining scores by firm size. A significant association *(p* 0.05) was found between the firm size and the self-sustaining index. As can be seen, large firms have a significantly higher self-sustaining score than small firms. Similarly, a significantly higher perceived success score (*p* 0.05) was associated with non-targeted initiatives than those specifically targeted at a certain cohort of the population, further illustrating the benefits of producing at scale. The degree to which legislation stipulated also seemed to have an impact on the perceived success of projects. For example, projects that were accompanied with mandatory fortification had a higher level (approaching significance at the *p* = 0.05 level) of perceived success among respondents than voluntary projects (data not shown). No differences were found by region. It should also be noted that a significant association (*p* 0.05) was found with the level of perceived success of the project and the degree to which it was self-sustaining. For example, those respondents that indicated projects were self-sustaining had a significantly higher perceived success index than for those respondents that had indicated otherwise (Figure 3). No significant differences were found with respect to the region and the respective indexes.

### 3.3. Standards and Type of Business Models (“Policy and Regulation” in Figure 1)

**Figure 4:**
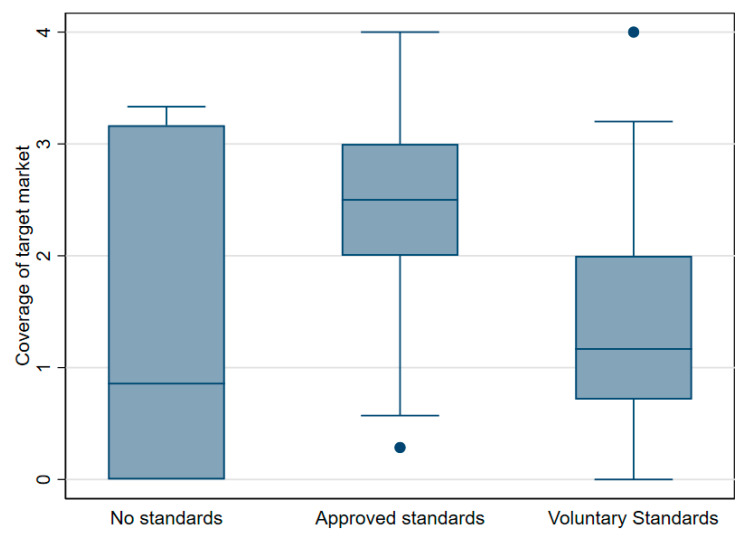
Box-plot showing a level of standards enacted and the coverage of target market (coverage of the target market: 0 = 0%—no production at the end of the project; 1 = 0–33% some—limited sustainable production achieved; 2 = 33–66% much—some markets supplied, but not all; 3 = 66–100% most—many markets receiving and using fortified food, but some gaps; and 4 = 100%—all domestic food of this type fortified with no subsidy). Note: the outside values are illustrated by dots. Adjacent line = upper adjacent value whiskers. The 75th percentile = the upper hinge box. The median value = dark line and the 25th percentile = lower hinge whiskers adjacent line.

Figure 4 highlights the importance of approved standards in relation to the overall coverage of the target market. Standards in this context refer to the quantity and quality of fortification and how this is assured and/or regulated. The degree to which standards are mandatory and enforced may be related to the business model adopted for fortification. A significant association (*p* 0.05) was found with the level of standards and the overall share of the target market. Approved standards clearly have a higher share of the target market (in this case, the product defined as available and being consumed by the target market) than voluntary/no standards. Moreover, a significant association was found between the quantity fortified by the firm/project and the level of overall compliance (*p* 0.05). The implementation of a business plan before the project/whilst the project was being implemented did not show any significant bearing on the perceived level of success compared to those that had never had a business plan in place. However, both groups which had implemented a business plan before the project commencement or during project implementation did have a higher mean score for both the success and self-sustaining indexes (See Table 3). No significant differences were found by business model either though models focusing on inter-level coordination (i.e., involving a specific collaboration between two levels in a value chain, e.g., processors and retailers) and a large food business (focused on large food businesses) had the highest perceived success scores (data not shown).

### 3.4. Premix Importation and Challenges (“Tradable vs. non Tradable” in Figure 1)

**Figure 5:**
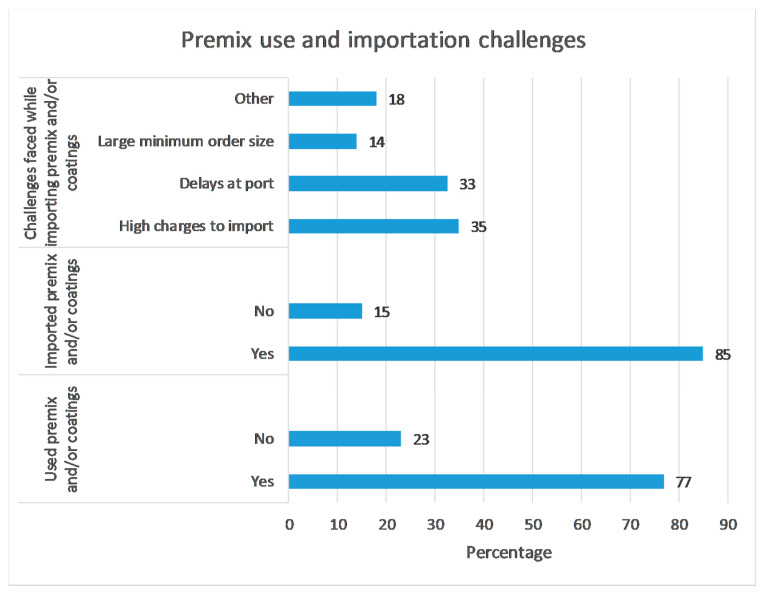
Premix/coatings usage, importation, and challenges by percentage.

Figure 5 highlights the premix usage among stakeholders and the level of reliance on imports. A high proportion of projects relied on premix and/or coating (coating technology is defined as the micronutrient premix which is added to rice kernels in a liquid fortificant mix—waxes and gums are usually used to allow for the micronutrient layer/layers to ‘fix’ to the rice grains [3]) (77%) with the overwhelming majority reliant on imports (85%). Challenges involved the high costs (charges involved) and delays at the port which significantly affect usage (Figure 5).

Table 4 shows the level of compliance to testing that takes place post-mix and the location. Though not always clearly compliant, there seems to be a good level of testing, whether in-factory or ex-factory (post-mix refers to the stage after the premixed fortificants have been added to the vehicle) to maintain the level of quality. Interestingly, the in-factory testing score also correlated significantly with the self-sustaining index (*p* 0.05) though ex-factory testing does not, which indicates that some level of post-mix testing is required to ensure programme sustainability. This points to the importance of investment in testing facilities that are within the ambit of the producers’ establishment and away from reliance on third-party testing laboratories. Further investigation of what factors drive a positive correlation between in-factory testing and sustainability could be an important further research question.

### 3.5. Stakeholders’ Views on Reasons behind Successful Projects

Table 5 presents the views of stakeholders regarding the reasons behind the success of fortification initiatives they have been involved with, which have been grouped under various themes and sub-themes. The main themes identified are broadly grouped under government support and private sector involvement. Legislation, enforcement and government subsidy are the sub-themes identified under government support. For example, legislation consisted of enabling standards and regulation, and mandating the involvement of the private sector players, whilst enforcement involved, e.g., monitoring/lab testing. Government subsidies in the form of training and/or equipment were also mentioned as reasons behind successful projects (Table 5). Private sector involvement through buy-in and attendance of training/sourcing of equipment were additional reasons provided for successful projects. This overlaps with the government subsidy theme relating to training and equipment (Table 5).

## 4. Discussion

The results have highlighted the importance of buy-in from the private sector in relation to industrial fortification initiatives. Clearly, the size of the firm involved in the fortification initiative has a bearing on sustainability (Figure 2) given the ability of larger firms to take advantage of economies of scale [18]. Large-scale industrial fortification has usually been successful where few firms reside and production is centralised [2]. Thus, if scale/replicability is needed, the incorporation of medium and larger sized firms seems to be an important contributor to success/sustainability, especially with regards to cereals [19]. However, the higher self-sustaining scores for medium-small firms compared to medium sized firms (Figure 2) indicates that medium-smaller sized firms may also play an important role in fortification initiatives. For example, maize production is highly fragmented in much of Tanzania, with a high proportion of maize flour being consumed in rural areas being produced by small or medium sized millers in these regions. Whilst compliance and quality assurance are still critical issues, options for increasing the scale of fortification have been found. Sanku is one example of an initiative supporting small to medium sized maize flour milling through use of an innovative dosifier. Sanku is able to monitor the miller’s use of the dosifier remotely (including monitoring the amount of premix), and is able to provide maintenance assistance by visiting the mill if the dosifier requires repair (Sanku, personal communication, 2020).

We also found that the legislation level also has a positive association with perceived success among stakeholders. Previous research has also indicated that the importance of mandatory fortification especially in relation to the large-scale fortification of cereals [7,19]. The enactment of mandatory fortification legislation may be particularly useful where voluntary fortification is currently pursued and leading to impasse. For example, Minimex (a company based in Rwanda) have initiated the voluntary fortification of maize flour but only a tiny proportion (10%) of their produce is fortified given it competes primarily with unfortified maize (Minimex, personal communication, 2020). Similar issues were faced in Uzbekistan when initiating wheat flour fortification. Whilst a number of state-run mills initiated fortification, private mills were not required to do so. In response, mandatory fortification legislation stipulated that all mills (state-run and private) must meet fortification standards in order to receive annual production permits [5]. However, the legislation enacted in Uzbekistan still seemed to be silent on imported unfortified produce. Morocco, for example, enacted legislation which stipulated that fortification of wheat flour is compulsory whether produced domestically or imported [5]. Thus, to be effective, legislation needs to adapt to consumption patterns and market trends.

The importance of legislative measures is further reinforced by the strong relationship between approved standards and the quantity of fortified/share of the target market. (Figure 4). A positive correlation was also found between the level of standards and overall coverage of the target market. Thus, this illustrates that stakeholders invariably believe that achieving an increased market share of the fortified produce is strongly related to the level of compliance. It is estimated, however, that compliance with standards can be as low as 50% in many LMICs (or even lower in some cases) and calls on governments to improve inspection and enforcement have been voiced [1,20]. Similarly, fortification in accordance with national standards is widely regarded as an essential component of overall success [2]. This is reinforced by the views of stakeholders in this study as they identified standards, monitoring/lab testing and enforcement/regulation as important contributors to success (Table 5).

Whilst we found the importance of enforceable standards as a priority, this necessitates adequate monitoring mechanisms including the improvement of the capacity of public laboratories to identify non-compliance [20]. In lieu of this, we found a positive relationship between in-factory testing and the self-sustaining index which also underscores the need for testing facilities at the producer level to ensure programme sustainability. Whilst there are challenges in doing so, including the lack of locally available testing devices and reagents, a number of ‘rapid-test kits and low-cost technology options’ are now on the market and being utilised [2]. Similarly, challenges to fortification were found relating to premix importation which included delays and high costs (Figure 5) which is confirmed by similar findings that have explored the topic [7,20]. In addition, to improve compliance and the overall standards of fortified produce, reducing nutrient losses related to premix will be particularly important. For example, nutrient losses occurring during storage have been found for premix batches tested in Kenya, thus more needs to be done to reduce such losses occurring across the supply chain [21]. Options such as tax incentives to encourage the procurement of premix and coordination of regulatory systems which includes the premix purchased in parallel for multiple food vehicles have been suggested to improve efficiency and oversight [2].

Likewise a “systems” approach to monitoring has been suggested, which will help to identify underfortified products earlier along in the value chain, including the onus on firms to improve record keeping and safety (allowing food inspectors to play more of a validation role) by employing principles of Good Manufacturing Practices (GMP) and Hazard Analysis/Quality Analysis and Critical Control Points (HACCP/QACCP) [20]. Improving the capacity of producers to internally monitor themselves has also been identified as key to ensuring standards are maintained [22].

## 5. Conclusions

This study has sought to unpack the ‘business model’ for fortification initiatives, to identify key drivers of success and constraints faced by stakeholders in LMICs. Our findings suggest that for a successful business model to reach its ‘target market’, the recommendation emerges as follows: greater coverage of fortification seems more successful at scale and with mandatory legislation and standards that are enforced. Clearly, different approaches may be needed depending on the vehicle chosen, consumption patterns and the desired target market. Whilst we did not find any significant differences between regions, other authors [15] have suggested that specific country groupings exist in relation to the scale/coverage of fortification initiatives. For example, fragile states where there is weak governance/lack of an enabling environment limits high coverage rates, as is the case in countries where awareness among key stakeholders and the strengthening of capacity needs to take place [15]. As found in previous research, however, larger sized firms play an important role with respect to the industrial fortification of cereals [2,7,19]. Careful consideration is needed to create legislation that accounts for factors, such as unfortified produce being imported/domestically produced. Moreover, in order for fortification initiatives to be properly implemented, a plan for sustainability needs to be built into program design a priori [5]. Given stakeholders felt business models involving a large food business (which arguably may be less prone to failure) and projects where a business plan was in place (prior to the commencement of the project or during the project) had higher perceived sustainability scores among stakeholders reinforces this.

Our qualitative analysis also indicated that stakeholders believe some level of subsidy is required, in the form of equipment/training or support with premix procurement. The adequate supply of premix was also raised by stakeholders as a key contributor to success. Likewise, stakeholders identified relevant standards, appropriate testing and enforcement/regulation as important contributors to success (Table 5). This is consistent with Luthringer et al., 2016 [20] who identified seven ‘broad reaching’ recommendations to improve the overall level of fortification compliance. These included enforcement, legislation, adequate human and financial capacity (including training, equipment etc.) and leadership, i.e., budgetary support from the government. Other authors have also highlighted legislative issues to be of paramount importance to the success of fortification initiatives; for example, the inclusion of detailed protocols including defined roles and responsibilities across agencies and clearer testing and sampling to be integrated into standards/regulation [22,23]. 

The findings of this study indicate a need for more in-depth evidence on delivery mechanisms based on specific case studies (food vehicles) of practical experience across a wide sample of countries and business cases. The discourse on business models for fortification currently lacks a typology that is accepted across the field. The characterisation of business types would improve future research considerably. More evidence is also needed to show how fortification models transition from externally supported to fully self-financing. Few examples of this transition were identified in this study.

## Figures and Tables

**Table 1:** The relationship between business context, hypothesised drivers of business success and failure, and fortification business contexts.

Questionnaire Theme	Context	Potential Drivers
Background information: respondent type, business example, vehicle, geography, fortification type, fortificant(s)	Objectives Viability model	Public vs. private Scale Governance and coordination
Business form: business scale, legislation level, market and targeting	Finance Technology Incentive structure	Scale Voluntary vs. mandatory Mature vs. start-up Output vs. process orientation
Technology: premix and tech importation challenges	Technology Policy and regulation	Tradable vs. non-tradable
Standards, regulation and business models	Policy and regulation	Voluntary vs. mandatory

**Table 2:** Survey variables and description.

Variables	Description of Variable/Code in Survey
Project name	Name of the project/firm involved in fortification
Region	Continent and country of the project were recorded
Commodities fortified (these were then disaggregated by type (e.g., wheat flour and maize flour, etc.)	1 = Fortified cereals and flours 2 = Fortified complementary foods 3 = Biofortified seeds and crops 4 = Other
Source of funding	1 = Donor 2 = Private investment, 3 = Public funding 4 = Other
Source of secondary funding	1 = Donor, 2 = Private investment 3 = Public funding 4 = Other
Legislation	1 = Voluntary (e.g., business can add or not), 2 = Mandatory (e.g., business must add by law) 3 = Other
Standards	1 = Approved standards 2 = Voluntary standard 3 = No standard
Coverage of the target market	1 = 0%—no production at end of project 2 = 0–33% some—limited sustainable production achieved 3 = 33–66% much—Some markets supplied, but not all 4 = 66–100% most—Many markets receiving and using fortified food, but some gaps 5 = 100%—all domestic food of this type fortified with no subsidy
Size of firm (number of employees)	1 = Large firm = 250 2 = Medium firm = 50 250 3 = SME = 10 50 4 = Small firm 10 5 = Other
Type of business model	1 = Value chain level collaboration (involving collaboration by different actors in the same value chain) 2 = Project-level partnerships (involving a specific project with different actors) Industry-level alliances (involving all actors in a particular sector e.g., millers) 3 = Multi-stakeholder platforms (involving actors at different stages and scales in a combination, e.g., processors and regulators) 4 = Inter-level coordination (involving a specific collaboration between two levels in a value chain e.g., processors and retailers) 5 = Small business food processing (focused on smaller food businesses) 6 = Large food business (focused on larger food businesses) 7 = Government feeding programme (government organised and funded programmes) 8 = Other
Success index	Perceived level of success of the fortification initiative: 1 = Failure 2 = Too early to tell 3 = Success.
Self-sustainability index	Perceived level of the sustainability of the fortification initiative: 1 = Model no longer functional or requires 100% subsidy 2 = Most from public subsidy 3 = Most from sales; 4 = 100%self-sufficient
Premix investment	1 = Yes 2 = No
Premix importation	1 = Yes 2 = No
Premix import challenges	1 = Delays at port 2 = High charges to import 3 = Large minimum order size 4 = Other
Business plan	1 = Plan before project 2 = Plan during project 3 = No business plan
Post-mix testing	In-factory testing and ex-factory testing Scored from 1—not available; to 5—fully functional and available
Standards infrastructure	National standards, conformity, traceability, and laboratories accreditation Scored from 1—not available; to 5—fully functional and available

**Table 3:** Mean and SE of the business plan implementation by the success index and self-sustaining index (success index: 1 = failure; 2 = too early to tell; 3 = success—self-sustain index: 1 = model no longer functional or requires 100% subsidy; 2 = most from public subsidy; 3 = most from sales; 4 = 100% self-sufficient).

Variable Name	N	Mean Success Score	Standard Error	Mean Self-Sustaining Score	Standard Error
Plan before project	25	2.64	0.14	3.25	0.21
Plan during project	14	2.42	0.19	2.92	0.29
No business plan	8	2.42	0.37	2.42	0.39

*p* values no significant difference. Note: some respondents did not respond to this question/N/A.

**Table 4:** Mean scores for post-mix testing/standards infrastructure (scored on scale, i.e., from 1—not available; to 5—available and fully functional).

Testing/Standards	Type of Standards or Testing Applied	Mean	N	Std. Dev.
Post-mix testing	In-factory testing	3	50	1.69
	Ex-factory testing	2.96	47	1.52
Standards infrastructure	National standard available and applied	3.58	52	1.55
	Conformity assessed by sampling, inspection, testing and certification	3.3	50	1.54
	Traceability system in place	2.88	48	1.44
	Laboratories and certification bodies accredited to international standards	3.45	51	1.53

**Table 5:** Reasons for the success of fortification projects by theme (N = 30).

Theme	Sub-Theme	Description of Themes Expressed by Respondents
Government support (e.g., law/policies and monitoring)	*Legislation* *Enforcement* *Subsidy*	Memorandum of understanding between government and industry/national strategy (N = 3) Enabling standards and regulations/mandatory inclusion by all players in the industry made mandatory by the government, chosen food vehicle is commonly consumed. (N = 4) Monitoring Lab testing capacity. (N = 3) Political will/enforcement to ensure quality monitoring. (N = 3) Machines, premix equipment supplied by government and/specialist training on milling. (N = 4)
Private sector involvement	*Buy-in* *Equipment/training*	Willingness of millers to adopt fortification. Effective leverage of industry association. (N = 3) Capacity strengthening support of stakeholders including industry support with social marketing and branding (N = 2). Public–private partnerships (PPP) (N = 5). Adequate supply of vitamin A premix, equipment. data generation, (N = 4)

Note: some respondents gave multiple responses.

## Appendix A

**Table A1:** Key characteristics of the fortification initiatives and measures of success.

Characteristics of Fortification Initiatives	Success Score	Self-Sustainability Score	Target Market
**Legislation**			
Mandatory vs. voluntary	Ns	Ns	Ns
**Target**			
Non-targeted vs. targeted	X	X	Ns
**Scale**			
Size of firm	Ns	X	Ns
**Standards**			
Approved/voluntary/no standard	Ns	Ns	X
**Testing**			
In-factory testing ex-factory testing	Ns Ns	X Ns	Ns Ns
**Business plan**			
E.g., Business plan before or at implementation vs. no business plan	Ns	Ns	Ns
**Type of Business model**			
Value chain level vs. inter-level coordination vs. large scale, etc.	Ns	Ns	Ns
**Region**	Ns	Ns	Ns
**Success**		X	Ns

Ns = non-significant X = significant *p* 0.05.
